# Post-Flowering Nitrate Uptake in Wheat Is Controlled by N Status at Flowering, with a Putative Major Role of Root Nitrate Transporter NRT2.1

**DOI:** 10.1371/journal.pone.0120291

**Published:** 2015-03-23

**Authors:** François Taulemesse, Jacques Le Gouis, David Gouache, Yves Gibon, Vincent Allard

**Affiliations:** 1 INRA, UMR 1095 Génétique Diversité et Ecophysiologie des Céréales, Clermont-Ferrand, France; 2 Université Blaise Pascal, UMR 1095 Génétique Diversité et Ecophysiologie des Céréales, Aubière, France; 3 Arvalis-Institut du Végétal, Service Biotechnologies, Boigneville, France; 4 INRA, UMR 1332 Biologie du Fruit et Pathologie, Villenave d’Ornon, France; Purdue University, UNITED STATES

## Abstract

In bread wheat (*Triticum aestivum L*.), the simultaneous improvement of both yield and grain protein is difficult because of the strong negative relationship between these two traits. However, some genotypes deviate positively from this relationship and this has been linked to their ability to take up nitrogen (N) during the post-flowering period, regardless of their N status at flowering. The physiological and genetic determinants of post-flowering N uptake relating to N satiety are poorly understood. This study uses semi-hydroponic culture of *cv*. Récital under controlled conditions to explore these controls. The first objective was to record the effects of contrasting N status at flowering on post-flowering nitrate (NO_3_^-^) uptake under non-limiting NO_3_^-^ conditions, while following the expression of key genes involved in NO_3_^-^ uptake and assimilation. We found that post-flowering NO_3_^-^ uptake was strongly influenced by plant N status at flowering during the first 300–400 degree-days after flowering, overlapping with a probable regulation of nitrate uptake exerted by N demand for growth. The uptake of NO_3_^-^ correlated well with the expression of the gene *TaNRT2*.*1*, coding for a root NO_3_^-^ transporter, which seems to play a major role in post-flowering NO_3_^-^ uptake. These results provide a useful knowledge base for future investigation of genetic variability in post-flowering N uptake and may lead to concomitant gains in both grain yield and grain protein in wheat.

## Introduction

Although grain yield (GY) remains the main breeding priority for wheat breeders, grain protein concentration (GPC) is another important priority, as GPC is a key element of wheat end-use value [1,2] and thus determines both the price paid to the farmer per tonne and also the opportunity for achieving export quality. However, the strongly negative relationship between GY and GPC [3,4] complicates the simultaneous improvement in these two traits, as increases in GY are generally detrimental to GPC. A classic agronomic strategy for obtaining high GY with adequate GPC in high-input agriculture is based on growing genotypes having strong yield potential, coupled with fertilisation practices that favour high GPC. In particular, delaying the last nitrogen (N) fertiliser application at heading, allows the low GPC, potentially associated with high GY, to be increased to an acceptable level. However, this approach is increasingly being questioned in a world where there is increasing pressure to reduce N inputs, both because of its rising cost and also because of a rising awareness of the environmental damage resulting from excessive N use in agriculture [5].

Monaghan *et al*. [6] proposed an alternative strategy for improving GPC without decreasing GY, thus breaking the negative GY-GPC relationship. This strategy originates from the observation that some genotypes exhibit robust deviations (whether positive or negative) from the regression of GPC on GY. This deviation, grain protein deviation (GPD), has a strong genetic basis [4,7] and has interesting potential as a breeding criterion. Based on this proposition, some studies have focused on identifying the physiological basis of genetic variability in GPD. In particular, Bogard *et al*. [8] showed that GPD was highly correlated with post-flowering N uptake regardless of plant N status at flowering. This led to the hypothesis that GPD could be conditioned by a putative genetic variability in the control of N satiety, that would allow some varieties to absorb more N after flowering. Testing of such a hypothesis demands a better understanding of post-flowering N uptake in wheat and its regulation by plant N status.

Grain N originates from two distinct sources: remobilisation from vegetative organs of N assimilated pre-flowering, and post-flowering uptake of N from the soil. The relative contributions to grain N of these two sources are negatively correlated [6,8,9], and are strongly dependent on environmental factors [10]. Typically, for wheat under field conditions, post-flowering N uptake contributes between 5 and 40% to total grain N [8,11–13]. The fraction depends also on growing context and, to a lesser extent, on genotype. A number of experiments have been carried out on wheat and barley under controlled conditions to study N uptake, including during the post-flowering period. These offer interesting understandings, such as of effects of N availability on the dynamics of biomass accumulation and N concentration [14], of N uptake dynamics under N-limiting conditions [15], of root N uptake capacity during the post-flowering period [16], and of the fate of the N taken up post-flowering [17]. Nevertheless, little is known of the kinetics of post-flowering N uptake, or of the associated control mechanisms. New information is needed at both the whole plant scale and at the molecular scale. In the latter case, the conservation of the metabolic pathways associated with both N uptake and N assimilation in higher plants, offers useful tools for studying post-flowering N uptake at the molecular scale.

Several recent reviews provide an extensive view of known mechanisms of N uptake and assimilation [18–21]. Uptake of N appears to be regulated mainly by plant demand [22,23]. However, a major area of continuing discussion among authors centres on the nature of the signal for N satiety. This has been proposed to be: (a) NO_3_^-^ [24–26] or (b) circulating amino-acids such as glutamine [27,28]. The principal forms in which N is taken up from the soil by plants are as nitrate (NO_3_^-^) and as ammonium (NH_4_^+^), with NO_3_^-^ being preferred under aerobic conditions [29]. In higher plants, NO_3_^-^ uptake involves two distinct systems classified according to their affinities for NO_3_^-^. The low-affinity transport system (LATS) is predominant when N concentrations ([N]) in the medium are high, with a K_m_ constant in the mM range. The high-affinity transport system (HATS) operates under low [N] conditions, with a K_m_ in the μM range [19].

LATS is coded by the NRT1 gene family which has 53 known members in *Arabidopsis thaliana* while HATS is coded by the NRT2 gene family which has seven members in *A*. *thaliana*. Among the LATS family, *AtNRT1*.*1* (*CHL1*) is the most studied, and both it and *AtNRT1*.*2* are expressed in the root epidermis and participate in NO_3_^-^ uptake [30]. Meanwhile, *AtNRT1*.*1* is a dual-affinity transporter being both constitutive and inducible, and it is involved in NO_3_^-^ uptake under both high- and low-NO_3_^-^ concentrations [31]. Moreover, it has recently been shown that *AtNRT1*.*1* can transport both NO_3_^-^ and/or auxin depending on the external concentrations of NO_3_^-^ [32]. In rice, *Oryza sativa*, *OsNRT1* is the homolog of *AtNRT1*.*1* and has been cloned and characterised as a low-affinity NO_3_^-^ transporter which is expressed constitutively in the root epidermis [33]. Based on sequence homology approaches, a number of other putative NRT1 family members (homologous to the *A*. *thaliana* NRT1 family) have been found in a range of species including: *Cucumis sativus*, *Oryza sativa*, *Zea mays*, *Sorghum bicolor* and *Brachypodium distachyon* [34,35]. Although homologues of most members of the *A*. *thaliana* NRT1 gene family probably exist in wheat, to our knowledge, only one has been studied [36].

The two main genes expressed in *A*. *thaliana* roots coding for HATS are *AtNRT2*.*1* and *AtNRT2*.*2*. Of these, AtNRT2.1, in interaction with an AtNAR2 protein [37] accounts for about 75% of the high-affinity NO_3_^-^ uptake [38]. These genes are induced by low NO_3_^-^ concentration ([NO_3_^-^]) and repressed by high [NO_3_^-^] and so belong to the group of inducible HATS. The HATS NO_3_^-^ uptake system is also implicated in plant responses to N starvation. Expression of *AtNRT2*.*1* increases rapidly when NO_3_^-^ is supplied to N-starved plants but later decreases if the provision of NO_3_^-^ is maintained [39]. Putative homologues of the main HATS coding genes in maize are *ZmNRT2*.*1* and *ZmNRT2*.*2*. These were detected by the sequence homology approach [34] and were later studied under hydroponic conditions where they seem to play a key role in N uptake even at [NO_3_^-^] higher than 1 mM [40]. The orthologue of *AtNRT2*.*1* in wheat is *TaNRT2*.*1*, and this has been characterised and its expression profiles suggest that it too belongs to the inducible HATS [41]. To our knowledge, *TaNRT2*.*1* is the only member of the *NRT2* gene family currently characterized in wheat.

Following its uptake, NO_3_^-^ is reduced to nitrite (NO_2_^-^) by nitrate reductase (NR) and the NO_2_^-^ is subsequently reduced to ammonium by nitrite reductase (NiR) [42]. A loop between glutamine synthetase 2 (GS2) and the two forms of glutamine 2-oxoglutarate amino transferase (GOGAT), NADH-GOGAT and Fd-GOGAT, finally integrate the N into amino acids [43]. Genes involved in N reduction and assimilation have been investigated in wheat [44–46].

The objective of our study was to provide a clearer view of post-flowering NO_3_^-^ uptake dynamics in wheat in relation to the N status at flowering. To study the uptake of NO_3_^-^ in detail, a semi-hydroponic approach under controlled conditions was established because of its susceptibility to environmental factors. Wheat plants (cv. Récital) were grown under four pre-flowering conditions with contrasting levels of [NO_3_^-^]. At flowering, all conditions were altered to be NO_3_^-^ non-limiting, allowing plants to fully exploit their NO_3_^-^ uptake potentials. The use of ^15^N pulse labelling allowed detailed recording of the dynamics of post-flowering NO_3_^-^ uptake and relative quantification of the expression of the main genes involved in both NO_3_^-^ uptake and assimilation and enabled links to be observed between gene expression and NO_3_^-^ uptake.

## Materials and Methods

### Plant material

Selected wheat grains (55 to 60 mg) of cv. Récital were sown in germination trays filled with compost and placed in a heated greenhouse at 20°C for 14 days. After emergence, plants were vernalised for six weeks in a plant growth chamber (6°C, 8 h photoperiod, light intensity 350 μmol PAR m^-2^s^-1^). After vernalisation, the roots were washed free of compost and plants were transplanted into PVC tubes (7 cm diam, 60 cm high) filled with a perlite:sand mixture (1:1, v:v) for semi-hydroponic culture. The lower end of each tube was closed with a perforated cap to contain the substrate while allowing excess nutrient solution to drain. Two plants were transplanted into each tube and tubes were placed vertically in eight containers (64 tubes per container, container area 0.49 m^2^). The resulting planting density (260 plants m^-2^) is comparable to that in the field under local agronomic practices.

Containers were placed in a growth chamber under a long-day photoperiod (16 h light at 20°C, 8 h dark at 18°C). Light intensity was 650 μmol PAR m^-2^s^-1^. Air temperature, humidity and light intensity at canopy level were recorded every 15 min using a CR1000 datalogger (Campbell Scientific, Logan, Utah, USA). Each tube was fitted with an automated micro-irrigation system, receiving one of four different nutrient solutions containing 1, 4, 7 or 10 mM NO_3_^-^ (the four nutrient treatments are referred to as N1, N4, N7 or N10, respectively). The nutrient solution compositions were adapted from Castle and Randall [47] (S1 Table). Two of the eight containers were irrigated with each of the nutrient solutions, with each tube receiving 66 ml of nutrient solution every three hours.

The flowering date (Zadok’s GS65) of each main stem was recorded. From flowering to maturity, all containers were irrigated with 66 ml every three hours of the same nutrient solution containing 10 mM of NO_3_^-^. Before each sampling, a subset of plants from each treatment (8 tubes per NO_3_^-^ pre-flowering condition) were submitted to 24 h of pulse labelling with ^15^N by watering with 66 ml every three hours of a 10 mM NO_3_^-^ nutrient solution enriched in ^15^N by 10%.

### Sampling protocol

Eight sampling dates were chosen during the post-flowering period. The first destructive sampling took place one day after flowering (GS65) and the remainder at approximately GS65+ 125, 250, 400, 500, 600, 800 and 1200 degree-days (DD) base 0°C. Each sampling was made at the same time of day (1–2 h after the start of the light period). On each sampling date, 16 tubes (32 plants) from each of the four NO_3_^-^ treatments were collected. Of these, four were used for physiological measurements and analysis of total nitrogen, four for gene expression and NO_3_^-^ analysis and the remaining eight for ^15^N analysis. Each tube (two plants) was considered a biological replicate.

### Physiological measurements and total nitrogen sample analysis

Physiological analyses were performed on four biological replicates. The processing of the two plants from each tube consisted first of counting the number of tillers and spikes per tube. The plants were then separated into six fractions; grains, chaff, stems (including leaf sheaths), green laminae, dry laminae, and roots. At stage GS65, in the absence of developed grains, ears were not separated into grain and chaff. All samples were oven dried to constant weight for 48 h at 80°C before dry weight (DW) measurement. Dry samples were ground to a fine powder using a ball mill and total nitrogen concentration was measured with the Dumas combustion method using a Flash EA 1112 Series CNS analyser (ThermoFisher Scientific, Illkirch, France).

### Analysis of ^15^N

Plants exposed to ^15^N pulse labelling were oven dried for 48 h at 80°C before DW measurement and ground before shipping to the UC Davis Stable Isotope Facility (Davis, CA, USA) for ^15^N abundance determination where samples were analysed using a PDZ Europa ANCA-GSL elemental analyser interfaced to a PDZ Europa 20–20 isotope ratio mass spectrometer (IRMS) (Sercon Ltd., Cheshire, UK). The ^15^N analyses were performed on eight biological replicates.

### Gene expression and NO_3_^-^ concentration assay sample preparation

The same samples were used to assay gene expression and NO_3_^-^ concentrations. Roots, stems and flag leaves of the two plants from each tube were pooled to form one biological replicate per organ, ground in liquid nitrogen, and stored at -80°C pending analysis.

### NO_3_^-^ concentration assays

Root, stem and flag leaf sub-samples (10 mg) were shipped to the Bordeaux INRA Metabolome Platform (https://www.bordeaux.inra.fr/umr619/RMN_index.htm; Bordeaux, France) for determination of NO_3_^-^ concentrations using a spectrophotometry method as described in Cross *et al*. [48]. The NO_3_^-^ assays were performed on four biological replicates.

### qRT-PCR experiments

Total RNA was extracted from three biological replicates of root, stem and flag leave samples from treatments N4 and N10 for the first seven sampling dates. Samples from the final sampling date were not extracted because of their advanced state of senescence. About 100 mg of frozen powder were used for each RNA extraction which was carried out using the Nucleomag 96 RNA kit (Macherey-Nagel, Düren, Germany) on the Biosprint 96 (Qiagen, Hilden, Germany). Total RNA samples were then purified with the NucleoSpin 96 RNA kit (Macherey-Nagel). The RNA quality was observed by migration in agarose gel. Reverse transcription of 1 μg of RNA was carried out with the iScript Select cDNA Synthesis kit (Bio-Rad, Richmond, California). The three steps—extraction, purification and reverse transcription—were carried out according to the manufacturer’s instructions.

Primer pairs were developed to quantify expression levels of *TaNRT1* (GenBank AY587264), *TaNRT2*.*1* (GenBank AF332214.1), *TaGS2* (GenBank DQ124212.1), *TaNR*, *TaNiR*, *TaFd-GOGAT* and *TaNADH-GOGAT* (whose partial sequence coming from Boisson *et al*. [44]) (S2 Table). All couples developed were generic and, thus, amplified three homeologous copies. The specificity of the primer pairs and their efficiency were validated between 85 and 100%.

The quantitative real-time PCR experiment (qPCR) was carried out on a LightCycler 480 system (Roche, Indianapolis, Indiana) with the LightCycler 480 SYBR Green 1 Master Kit. The qPCR program involved pre-incubation for 10 min at 95°C, followed by 45 cycles of amplification, each consisting of denaturation for 10 s at 95°C, followed by annealing for 15 s at 60°C and finishing with elongation at 72°C for 15 s. To ensure that single products were amplified, a melting curve was analysed at the end of each assay. Because of relatively low expression levels, the two NO_3_^-^ transporter genes were analysed with the cDNA template diluted to 1/8, while for all other genes the cDNA template was diluted to 1/20. qPCR was carried out using a 15 μl reaction volume containing 5 μl diluted cDNA, 7.5 μl SYBR green mix, 0.75 μl each of 10 μM forward and reverse primers and 1 μl of water. Relative expression was determined using the ΔCT method corrected for primer efficiency [49]. Results were normalised to the expression of two housekeeping genes, Ta54280 and Ta54948, selected from Paolacci *et al*. [50] (S2 Table) whose expression stability had already been validated under our experimental conditions.

### Statistical analyses

Results were analysed after conversion to a per-square-meter basis, using tube surface area. All statistical analyses were carried out using R v2.15.1 [51], and graphics were drawn using SigmaPlot v8.0.

### Nitrogen nutrition index

The N nutrition index (NNI) was calculated at flowering as the above-ground N concentration divided by the critical plant N concentration. The critical plant N concentration is defined as the minimum N concentration needed for maximum growth rate [52]. The critical N concentration was calculated using the equation of the critical N concentration curve for cereals proposed by Justes *et al*. [53].

## Results

### Effects of pre-flowering NO_3_^-^ treatments on plant morphology, biomass and N concentration

Four levels of NO_3_^-^ in the nutrient solutions were used until flowering (1, 4, 7 and 10 mM, respectively referred to as N1, N4 N7 and N10). These led to highly contrasting tiller numbers, biomasses and N concentrations at flowering (Table 1). Tiller number increased significantly with rising [NO_3_^-^]. Values lay between 522 tillers m^-2^ for N1, and 1371 tillers m^-2^ for N10, indicating a strong morphological response to rising N. Tiller number increased linearly with [NO_3_^-^] (r^2^ = 0.99, p = 0.005; data not shown), with an average slope of 92.5 tillers m^-2^ per mM of [NO_3_^-^]. Plant biomass ranged from 393 gDW m^-2^ for N1 to 1702 gDW m^-2^ for N10. Even though the biomass difference between plants at flowering from N4 to N7 was not significantly different (p = 0.168), a linear regression across the four [NO_3_^-^] treatments vs plant biomass did reveal a significant relationship (r^2^ = 0.95, p = 0.026; data not shown). This trend shows that all treatments lay within a range where [NO_3_^-^] was limiting for plant growth. Plant N concentration (plant [N]) at flowering increased strongly and linearly with nutrient [NO_3_^-^] (r^2^ = 0.99, p = 0.003; data not shown). The average slope was 0.183 percentage points of plant [N] per mM of NO_3_^-^.

**Table 1:** Tiller number, plant dry weight, plant N concentration, total plant N and NNI at flowering for the four NO_3_^-^ treatments.

NO_3_^-^ treatment	Tiller number (m^-2^) ±SE	Plant dry weight (g m^-2^) ±SE	Plant N concentration (%DW) ±SE	Total plant N (g m^-2^) ±SE	NNI ±SE
**N1**	522± 53 *d*	393± 33 *c*	1.512± 0.078 *c*	5.88± 0.30 *d*	0.50± 0.01 *d*
**N4**	784± 53 *c*	1100± 17 *b*	2.133± 0.062 *bc*	23.47± 0.83 *c*	1.12± 0.02 *c*
**N7**	1012± 63 *b*	1335± 149 *b*	2.710± 0.264 *ab*	35.08± 1.24 *b*	1.53± 0.07 *b*
**N10**	1371± 38 *a*	1702± 83 *a*	3.145± 0.161 *a*	53.15± 0.81 *a*	1.95± 0.09 *a*

Values are the means of four biological repetitions ± 1 standard error (SE). Statistically non-significantly different groups (Tukey multiple comparisons, p < 0.05) are labeled with the same lowercase letter. NNI = Nitrogen Nutrition Index.

All the above trends are supported by a calculation of NNI at flowering (Table 1), which shows a significant linear response to NO_3_^-^ (r² = 0.99, p = 0.005; data not shown). The average increase was 0.22 points per mM of [NO_3_^-^]. With regard to the critical nitrogen dilution curve developed for wheat [53], only N1 appeared to be limited by N status with NNI less than 1. This index was developed for field crops, so it should be used carefully under our conditions, nevertheless it clearly indicates a differential effect for the four [NO_3_^-^] treatments used in our study.

After flowering, all plants were exposed to the same, non-limiting 10 mM NO_3_^-^ nutrient solution. At maturity, the DW hierarchy between treatments was conserved (Table 2) although there was no significant difference between N7 and N10. The range of DW was fairly consistent with values of 1701, 2966, 4637 and 4822 gDW m^-2^ for N1, N4, N7 and N10, respectively. For plant [N] at maturity, despite no significant differences between N1, N4 and N10, there did seemed to be a trend for plants having been exposed to high pre-flowering N, to have less plant [N] at maturity. Plant [N] at maturity was 2.1 and 2.2%DW for the high-NO_3_^-^ treatments (N7 and N10, respectively) and 2.4 and 2.5% DW for the low-NO_3_^-^ treatments (N1 and N4, respectively).

**Table 2:** Plant dry weight, plant N concentration, grain yield, grain N concentration and harvest index at maturity for the four NO_3_^-^ treatments.

NO_3_^-^ treatment	Plant dry weight (g m^-2^) ±SE	Plant N concentration (%DW) ±SE	Total plant N (g m^-2^) ±SE	Grain yield (g m^-2^) ±SE	Grain N concentration (%DW) ±SE	Total grain N (g m^-2^) ±SE	Harvest index ±SE
**N1**	1701± 187 *c*	2.438± 0.061 *a*	41.40± 4.43 *c*	690± 103 *b*	3.050± 0.107 *a*	20.79± 2.45 *b*	0.44± 0.02 *a*
**N4**	2966± 251 *b*	2.452± 0.066 *a*	72.46± 5.59 *bc*	1051± 109 *b*	2.926± 0.054 *a*	30.73± 3.22 *b*	0.39± 0.01 *a*
**N7**	4637± 270 *a*	2.086± 0.043 *b*	96.73± 6.10 *ab*	1828± 90 *a*	2.549± 0.074 *b*	46.58± 2.49 *a*	0.44± 0.02 *a*
**N10**	4822± 375 *a*	2.242± 0.113 *ab*	108.79± 12.77 *a*	1944± 179 *a*	2.489± 0.097 *b*	48.72± 5.79 *a*	0.43± 0.02 *a*

Values are the means of four biological repetitions ± 1 standard error (SE). Statistically non-significantly different groups (Tukey multiple comparisons, p < 0.05) are labeled with the same lowercase letter.

Interestingly, the two groups of treatments described for plant [N] stood out for the agronomic variables of grain yield, grain N concentration (grain [N]) and total grain N. There were no significant differences in grain yield either between N1 and N4 (690, 1051 g m^-2^, respectively) or between N7 and N10 (1828 and 1944 g m^-2^, respectively). Probably due to the semi-hydroponic growth conditions, grain yields were high (equivalent to 69 and 194 q ha^-1^ for the two extreme treatments). Grain [N] values were 3.1 and 2.9%DW for N1 and N4, respectively and 2.5%DW for N7 and N10, respectively. There were strong and highly significant opposite-going effects of pre-flowering NO_3_^-^ treatment on grain yield and grain [N]. Grain yield and grain [N] were therefore negatively correlated (r^2^ = 0.99, p = 0.002). Total grain N (Table 2) was lower than total post-flowering N uptake (calculated by difference between total plant N at maturity, Table 2, and total plant N at flowering, Table 1), indicating that there was no net remobilisation of N from vegetative parts to the grains under our semi-hydroponic conditions. Harvest index lay between 0.39 and 0.44, which is close to values commonly observed under field conditions.

**Fig 1:**
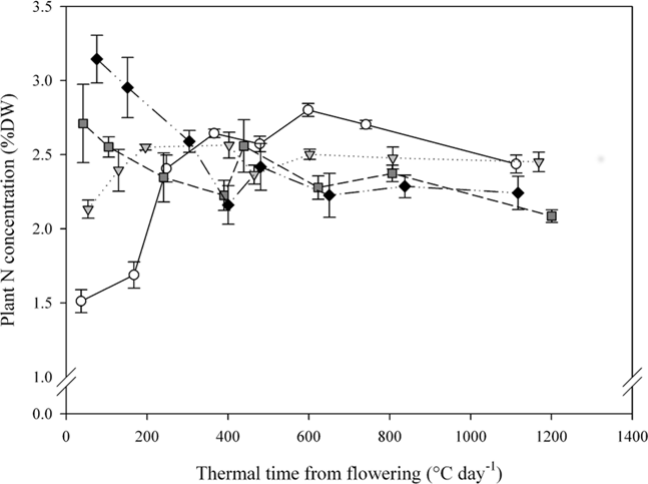
Plant N concentration dynamics during the post-flowering period for the four NO_3_^-^ treatments. Values are the means of four biological repetitions ± 1 standard error for N1 (open circles), N4 (grey triangles), N7 (dark grey squares) and N10 (black diamonds).

Starting from significantly different levels of plant [N] at flowering, plants from the four [NO_3_^-^] treatments quickly converged showing comparable [N] by GS65+300DD (Fig. 1). Convergence occurred between flowering and GS65+300DD, and led to plant [N] values between approximately 2.1 and 2.5%DW for all treatments. Interestingly, the convergence between treatments was reflected not only in an increase in plant [N] in the low-N pre-flowering treatments (N1 and N4) but also in a decrease in the high-N pre-flowering treatments (N7 and N10). During this phase, plants from N1 and N4 exhibited [N] increases of 59 and 20%, respectively, while plants from N7 and N10 exhibited [N] decreases of 13 and 18%, respectively. From GS65+300DD until maturity, plant [N] remained relatively stable for all treatments, varying between 2.0 and 2.7%DW.

### NO_3_^-^ uptake during the post-flowering period

**Fig 2:**
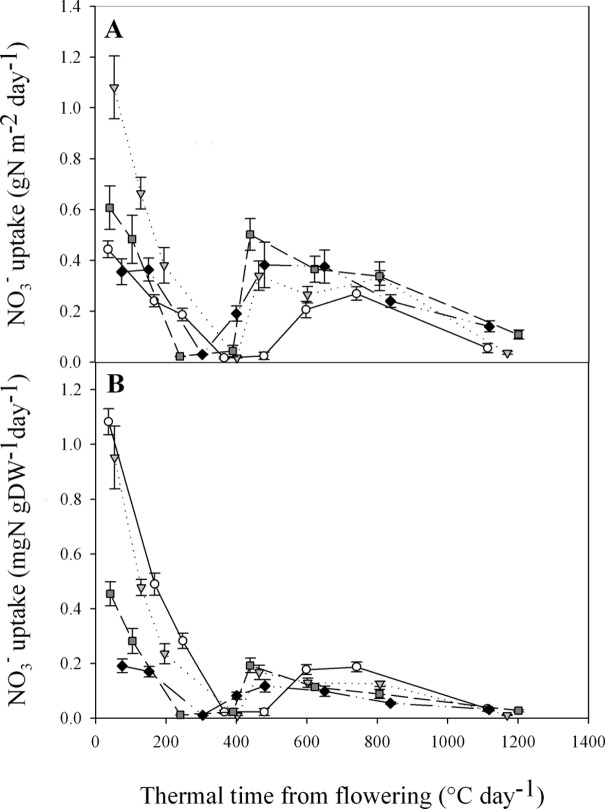
Plant NO_3_^-^ uptake dynamic during post-flowering period for the four NO_3_^-^ treatments per square meter (A) or per unit DW (B). Data were obtained by substitution of the previous nutrient solution by an identical one but labelled 10% ^15^N nutrient solution 24 h before sampling. Values are the means of eight biological repetitions ± 1 standard error for N1 (open circles), N4 (grey triangles), N7 (dark grey squares) and N10 (black diamonds).

The ^15^N pulse labelling allows precise measurement of the dynamics of plant NO_3_^-^ uptake during the post-flowering period. Fig. 2A shows that all treatments had strong NO_3_^-^ uptakes at flowering, with levels between 0.36 and 1.08 gNO_3_^-^ m^-2^day^-1^ depending on [NO_3_^-^] treatment. After flowering and regardless of [NO_3_^-^] treatment, NO_3_^-^ uptake showed a rapid decrease from flowering until a date between GS65+300DD and GS65+400DD where uptake stopped. NO_3_^-^ uptake then recovered rapidly to a substantial level between 0.2 and 0.5 gNO_3_^-^ m^-2^day^-1^ depending on [NO_3_^-^] treatment and then slowly declined until maturity. In addition to NO_3_^-^ uptake dynamics, these results also show that NO_3_^-^ uptake continued until late during the post-flowering period when plants were exposed to a non-limiting level of NO_3_^-^ in the nutrient solution.

When NO_3_^-^ uptake is normalised to plant DW, it is easier to interpret differences in NO_3_^-^ uptake between the four [NO_3_^-^] treatments (Fig. 2B). Although in terms of temporal dynamics, NO_3_^-^ uptake looks very similar either in absolute terms (Fig. 2A) or when normalised by DW (Fig. 2B), the latter representation shows that plants from N1 had strong NO_3_^-^ uptakes per unit DW (Fig. 2B). As expected, NO_3_^-^ uptake was strongly impacted by plant N status at flowering. Also at flowering, NO_3_^-^ uptake per unit DW was considerably larger for plant exposed to the low [NO_3_^-^] treatments pre-flowering. Relative to N10, daily fluxes of NO_3_^-^ uptake were 5.7-, 5- and 2.4-times higher for N1, N4 and N7, respectively. Furthermore, the normalisation of NO_3_^-^ uptake per unit DW allows better comparisons of the different periods of the temporal dynamic. Indeed, the direct effect of NO_3_^-^ uptake on plant [N] was much stronger at flowering than later during the post-flowering period (Fig. 2B). Lastly, the exact stage of NO_3_^-^ uptake recovery seems to differ between the [NO_3_^-^] treatments. The most obvious trend is that plants exposed to higher pre-flowering [NO_3_^-^], restarted uptake earlier than those exposed to lower pre-flowering [NO_3_^-^].

### Post-flowering dynamics of [NO_3_^-^] and NO_3_^-^ transporter gene expression in roots

The gene expression and NO_3_^-^ concentration measurements were carried out only on N4 and N10 plants because these represent a best compromise between a distinct N effect and non-extreme plant structures. Because of the strong correlation observed between roots and stems [NO_3_^-^] dynamic during the post-flowering period (r^2^ = 0.695, p<0.0001) (S1 Fig), and because flag leaves were not impacted by NO_3_^-^ treatment (S3 Table), the results presented in this section are based on root samples only. This specific focus on roots for [NO_3_^-^] was chosen to improve results clarity, given that roots and stems bring redundant information, and flag leaves seemingly bring no information on the differences on plant N status observed in the present study. Results from stems and flag leaves are however available as supplementary data (S2 Fig).

**Fig 3:**
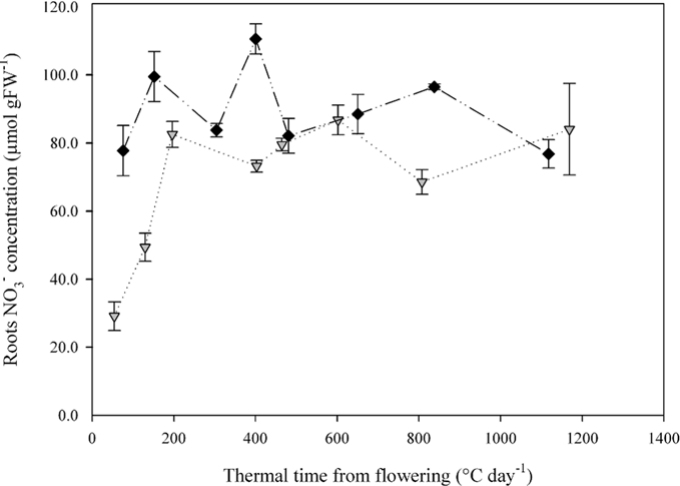
Roots NO_3_^-^ concentration dynamics during the post-flowering period for two contrasting NO_3_^-^ treatments (N4 and N10). Values are the means of four biological repetitions ± 1 standard error for N4 (grey triangles) and N10 (black diamonds).

Levels of root [NO_3_^-^] at flowering were strongly impacted by NO_3_^-^ treatment (Fig. 3). Indeed, at flowering, N4 plants exhibited root [NO_3_^-^] of 29.1 μmol gFW^-1^ whereas in N10 plants [NO_3_^-^] was 2.6-times higher (77.8 μmol gFW^-1^). After flowering, the two treatments rapidly converged to around 80 μmol gFW^-1^ after about 300DD. Despite some variability between sampling dates, [NO_3_^-^] remained relatively stable thereafter. The rapid convergence of root [NO_3_^-^] between the two treatments was caused mainly by a near doubling of [NO_3_^-^] in N4 during the 200 DD immediately following flowering (Fig. 3). The levels of [NO_3_^-^] in the roots were not significantly related to NO_3_^-^ uptake (data not shown).

**Fig 4:**
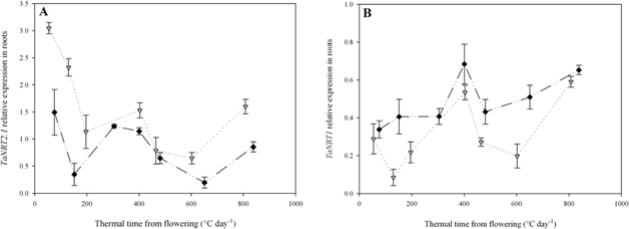
Relative expression patterns of *TaNRT2*.*1* (A) and *TaNRT1* (B) genes in roots from flowering (GS65) to GS65+800DD for two contrasting NO_3_^-^ treatments (N4 and N10). Values are the means of three biological repetitions ± 1 standard error for N4 (grey triangles) and N10 (black diamonds). Quantification was performed by qRT-PCR. Relative expression values were calculated using the ΔCT method corrected for primer efficiency, using *Ta54280* and *Ta54948* as internal controls.

In the two members of the main families of low and high affinity NO_3_^-^ transporters, *TaNRT1* and *TaNRT2*.1, post-flowering gene expression dynamics provides valuable information that increases our understanding of NO_3_^-^ uptake. For *TaNRT2*.*1*, the relative expression level at flowering depended on the pre-flowering NO_3_^-^ treatment (Fig. 4A) with its expression in N4 roots being twice that in N10 roots. Both treatments then exhibited a sharp decrease in expression of root *TaNRT2*.*1* between flowering and GS65+200DD. After that, the difference in expression between the two treatments became small, both showing an increase after 200DD and a further decrease between GS65+400DD and GS65+600DD. The last measurement point shows an unexpected increase in *TaNRT2*.*1* expression for both treatments but the more pronounced increase in N4 led to significant differences between N4 and N10.

The relative expression dynamics of *TaNRT1* in roots (Fig. 4B) differed from that of *TaNRT2*.*1*. First, expression of *TaNRT1* was low at flowering with no significant difference in relative expression levels between N4 and N10 at that time. After that, expression in N4 plants was consistently lower than in N10 plants, except at GS+400. Second, the expression patterns between the two genes are extremely different, with *TaNRT1* showing an increase in expression from flowering to GS65+400DD and then a decrease which was more pronounced in N4 than in N10. This was followed by an increase during the final measurement period in N4 which came into balance with expression in N10.

**Fig 5:**
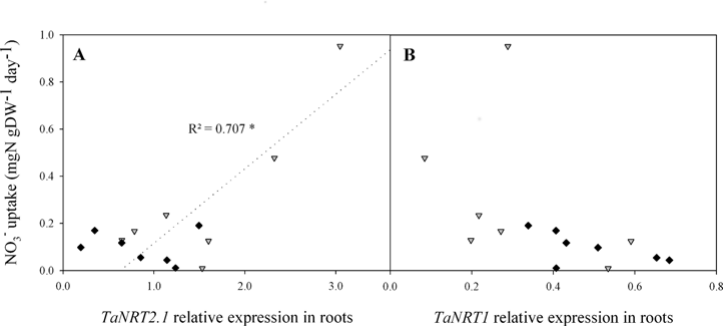
Relations between plant NO_3_^-^ uptake and relative expression levels of *TaNRT2*.*1* (A) and *TaNRT1* (B) in roots from flowering (GS65) to GS65+800DD for two contrasting NO_3_^-^ treatments (N4 and N10). Uptake data were obtained by substitution of previous nutrient solution by an identical one but labelled 10% ^15^N nutrient solution 24 h before sampling. Values are means of eight biological repetitions for NO_3_^-^ uptake and of three biological repetitions for gene expression for N4 (grey triangles) and N10 (black diamonds). Correlations are based on seven post-flowering sampling dates. Statistical analyses were by the Pearson correlation test. Gene expression quantification was performed by qRT-PCR. Relative expression values were calculated using the ΔCT method corrected for primer efficiency, using *Ta54280* and *Ta54948* as internal controls.

The level of NO_3_^-^ uptake per unit DW was significantly and positively correlated with *TaNRT2*.*1* expression in N4 (r^2^ = 0.707, p = 0.018; Fig. 5A) but not in N10 (r^2^ = 0.011). The variation in both variables was extremely small in the latter treatment relative to those in N4 plants. Conversely, *TaNRT1* expression was not significantly correlated with NO_3_^-^ uptake (r^2^ = 0.21 and 0.33 for N4 and N10, respectively; Fig. 5B).

**Fig 6:**
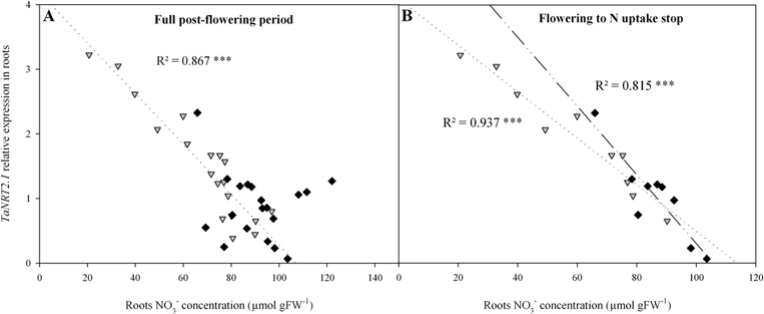
Relations between roots NO_3_^-^ concentration and relative expression level of *TaNRT2*.*1* in roots for two contrasting NO_3_^-^ treatments (N4 and N10) from flowering (GS65) to GS65+800DD (A) and from flowering (GS65) until NO_3_^-^ uptake stop (respectively GS65+400DD and GS65+300DD for N4 and N10) (B). Values are the three individual biological repetitions for seven post-flowering sampling dates for N4 (grey triangles) and N10 (black diamonds) (A) or respectively four and three sampling dates for N4 and N10 (B). Gene expression quantification was performed by qRT-PCR. Relative expression values were calculated using the ΔCT method corrected for primer efficiency, using *Ta54280* and *Ta54948* as internal controls. Statistical analyses were by the Pearson correlation test.

A strong negative correlation was observed between root [NO_3_^-^] and *TaNRT2*.*1* expression for N4 (Fig. 6A), while no significant relationship was detected for N10. Results from N10 plants were significantly impacted by three unexplained outliers originating from the GS65+400DD sampling date. Without these, the relation between the two variables would have been comparable under the two treatments.

In order to focus on a developmental phase when the grain sinks for N were weak, the relation between root NO_3_^-^ content and *TaNRT2*.*1* expression was also observed during the period between flowering and GS65+300DD. Here, the negative relationship between *TaNRT2*.*1* expression and root [NO_3_^-^] was highly significant for both treatments (r^2^ = 0.94 and 0.82 for N4 and N10, respectively, Fig. 6B).

### NO_3_^-^ reduction and assimilation network

To provide a biologically coherent description, correlations between the expressions patterns of the major genes involved in NO_3_^-^ uptake, the reduction and assimilation networks were examined pairwise according to the path of NO_3_^-^ assimilation. Root samples from N4 and N10 were observed independently. In the same way as for [NO_3_^-^], results presented for gene expression are based solely on root samples. This specific focus on roots was again retained for clarity, enabled by high positive correlations (0.61 < r < 0.81) observed between roots and flag leaves for all observed genes except *TaNADH-GOGAT* (S3 Fig). Expression dynamics in stems during the post-flowering period were however correlated to those observed in roots for *TaNR* only (S4 Fig).

**Fig 7:**
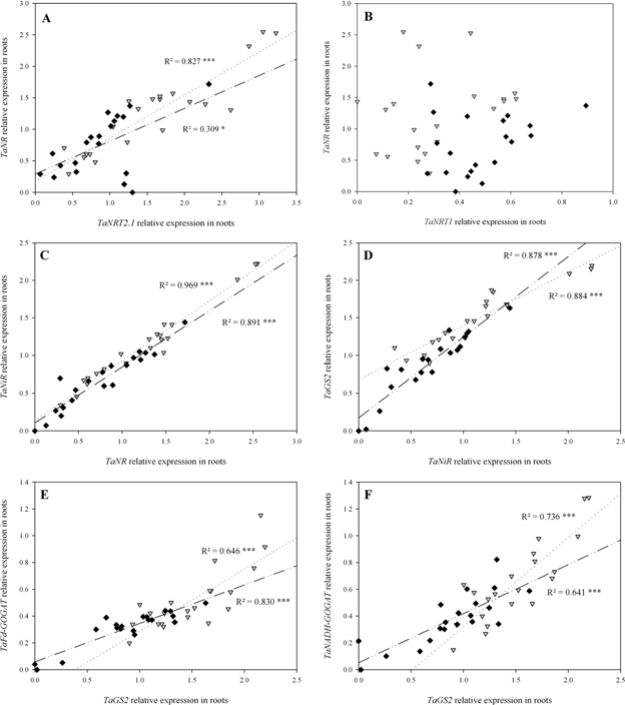
Correlations between relative expression patterns of the main genes involved in NO_3_^-^ uptake, reduction and assimilation in roots from flowering (GS65) to GS65+800DD for two contrasting NO_3_^-^ treatments (N4 and N10). Values originate from seven post-flowering sampling dates, each including three individual biological repetitions for N4 (grey triangles) and N10 (black diamonds). Relations are for (A) *TaNRT2*.*1* and *TaNR*, (B) *TaNRT1* and *TaNR*, (C) *TaNR* and *TaNiR*, (D) *TaNiR* and *TaGS2*, (E) *TaGS2* and *TaFd-GOGAT*, and (F) *TaGS2* and *TaNADH-GOGAT*. Quantification was performed by qRT-PCR. Relative expression values were calculated using the ΔCT method corrected for primer efficiency, using *Ta54280* and *Ta54948* as internal controls. Statistical analyses were by the Pearson correlation test.

There was a significant positive correlation between the expression patterns of the high-affinity NO_3_^-^ transporter family member *TaNRT2*.*1* and the nitrate reductase *TaNR* in both N4 and in N10 in roots (Fig. 7A). The lower r^2^ observed for N10 is explained mainly by the three outliers. Conversely, there was no obvious link between the expression of the low-affinity NO_3_^-^ transporter family member *TaNRT1* and *TaNR* either in N4 or in N10 (Fig. 7B). Subsequently, there was a strong positive correlation between *TaNR* and the nitrite reductase (*TaNiR*) expression patterns (Fig. 7C). Correlations were significant in both N4 and N10 between these two contributors to N reduction. Continuing to follow the path of N in the plant, significant positive correlations were observed between the expressions of *TaNiR* and glutamine synthetase 2 gene (*TaGS2*) in the two treatments (Fig. 7D). Lastly, correlations were observed between the expressions of *TaGS2* and two forms of glutamine oxoglutarate aminotransferase (*GOGAT*)—ferredoxin-dependent *GOGAT* (*TaFd-GOGAT*; Fig. 7E) and NADH-dependent *GOGAT* (*TaNADH-GOGAT*; Fig. 7F). At this level of N assimilation, expression patterns were positively and significantly correlated both in N4 and N10. The overall observation of correlations between the relative expression patterns of all these central network genes suggests a coordinated regulation of nitrogen metabolism with, probably, a limited involvement of *TaNRT1* under our experimental conditions.

## Discussion

The objectives of this study were to provide a detailed vision of post-flowering N uptake and to identify possible control mechanisms, using a prospective approach based on correlations between physiological traits, NO_3_^-^ uptake, NO_3_^-^ concentrations and expression level of key genes of the N metabolism. Clearly, the present work does not provide any functional dissection of the interaction between these physiological processes but highlights strong correlations between variables during post-flowering N uptake on wheat plants in realistic conditions in term of plant size, canopy structure and grain sink strength.

To achieve this, wheat plants were grown under four pre-flowering NO_3_^-^ treatments to create contrasting N statuses at flowering and monitor post-flowering NO_3_^-^ uptake under non-limiting NO_3_^-^ conditions. Results show that NO_3_^-^ uptake occurred until late grain filling. N status influenced NO_3_^-^ uptake rates at flowering and all treatments converged rapidly towards comparable levels of NO_3_^-^ uptake. Treatment differences in [N] at flowering decreased considerably under non-limiting NO_3_^-^ conditions. NO_3_^-^ uptake rate was correlated with *TaNRT2*.*1* expression, which in turn was correlated with *TaNR* expression. The main components of the gene network involved in NO_3_^-^ uptake, NO_3_^-^ reduction and N assimilation showed coordinated patterns of expression. Root [NO_3_^-^] levels were negatively correlated with *TaNRT2*.*1*, indicating that NO_3_^-^ could be a regulating element for *TaNRT2*.*1* expression and, so also of NO_3_^-^ uptake. Here, we will discuss: (i) the experimental conditions; (ii) the control of post-flowering NO_3_^-^ uptake; (iii) the putative impact of *TaNRT2*.*1* on NO_3_^-^ uptake and its putative regulation; and (iv) possible limiting factors for grain [N] under extreme conditions of NO_3_^-^ availability.

### Evaluation of growing conditions

Growing wheat in controlled hydroponic conditions allows nutrient availability to be closely regulated. Nevertheless, it is difficult to obtain nutrient availabilities fully comparable with those under field conditions. Previous studies [14–17,54] have developed fertilisation protocols for use in hydroponic culture that allow field N conditions to be mimicked with constant availability of growth-limiting levels of N. Here, a more classical approach was used to drive growth during the pre-flowering period. This involved four distinct NO_3_^-^ treatments and generated canopies of markedly contrasting structure. The level of [NO_3_^-^] in the nutrient solutions strongly influenced pre-flowering plant development, with increments of [NO_3_^-^] increasing tiller density linearly by about 90 tillers m^-2^mM^-1^, biomass by about 140g m^-2^mM^-1^ and [N] by 0.2 percentage points mM^-1^.

A further objective of the experimental setup was to apply NO_3_^-^ at excess levels during the post-flowering period in order to study the effects of N status at flowering on NO_3_^-^ uptake under non-limiting conditions. Although 10 mM [NO_3_^-^] in a nutrient solution is usually considered to be non-limiting for growth in *Arabidopsis* [21,55,56], and is usually the highest level N treatment in hydroponic wheat [36,57], it is difficult to be certain that this level was strictly non-limiting as we did not include treatments with even higher levels of [N]. Barneix [58] stated in a review that high N uptake rates, lead to high levels of N nutrition, but they inhibit leaf senescence and the remobilisation of N to the grain. In our study, the absence of significant net remobilisation of N from vegetative parts during the post-flowering period (the amount of N taken up was higher than the total in the grain for all treatments) suggests that NO_3_^-^ was indeed available in a sufficient quantity. This scenario differs from the 60 to 95% of N remobilisation usually observed in the field [8,11,13].

Therefore, it seems reasonable to infer that the NO_3_^-^ treatments used here led not only to highly contrasting stands at flowering but they further allowed the post-flowering growth to be non-limiting with regard to NO_3_^-^.

### Post-flowering control of NO_3_^-^ uptake

Under our semi-hydroponic, non-limiting NO_3_^-^ conditions, plants exhibited positive NO_3_^-^ uptake until late in grain filling. Similar observations have been reported for barley cultured hydroponically under N-limiting conditions [15] and for wheat [14,16,17,54], as well as for wheat under semi-controlled conditions, more similar to the field [59]. In these studies, N uptake also occurred until close to grain maturity, showing that these plants do not have an intrinsic physiological incapacity to take up N during grain filling. Compared to the NO_3_^-^ uptake rates in the present study, the lower rates generally observed in the field are thus unlikely related to an intrinsic incapacity of the plant but instead to some unfavourable conditions that affect either the availability of N in the soil or the plant’s demand for it.

In their hydroponic study on wheat, Oscarson *et al*. [16] showed that under N-limiting conditions, N uptake gradually decreases throughout the post-flowering period. However, under non-limiting conditions, we show that NO_3_^-^ uptake is far from constant from flowering to maturity. This leads to the hypothesis that NO_3_^-^ uptake is regulated by factors other than NO_3_^-^ availability. Information on N uptake dynamics in wheat under non-limiting conditions is scarce, particularly during the post-flowering period. Both Imsande and Touraine [60], and Feil [22] in a review based on both controlled conditions and field experiments hypothesised that N uptake reflects the internal demand of the crop and is not determined only by the external [N]. This idea also finds support in some more recent experimental studies [61,62] as well as in assessments of N uptake arising from wheat crop simulation models. These models, despite some calculation differences, calculate N uptake as governed by a balance between the plant’s needs to support growth and the availability of N in the soil [63]. Thus, under non-limiting N conditions, N uptake is potentially fully governed by the plant’s demand for N for growth.

If the present study confirms earlier findings on post flowering N uptake in wheat, it also provides a clearer view of the small-scale dynamics of the process. Post-flowering NO_3_^-^ uptake dynamics can be divided into three distinct phases. The first of these is from flowering to sometime between GS65+300DD and GS65+400DD (depending on treatment). Here NO_3_^-^ uptake declines considerably from its level at flowering. Next, in the second phase, NO_3_^-^ uptake almost ceases for a period of about 100DD. And last, in the third phase, a recovery of NO_3_^-^ uptake occurs which persists to maturity. Interestingly, each phase coincides with a physiological evolution in development, supporting the idea that NO_3_^-^ uptake could be strongly controlled by a growth demand for N.

The general decline in NO_3_^-^ uptake during the first phase is synchronous with a reduction in the sink strength of plant growth. Indeed, immediately after flowering, there are few new sinks for N as the photosynthetic apparatus is already fully developed, whereas the spikes are not yet into a phase of rapid biomass increase. The main biomass increment occurring during this phase is driven by post flowering stem elongation. The stem is a structural tissue that is relatively poor in N compared with, say, a leaf. Both the upper internode and the ear peduncle are known to continue extension for approximately one week after anthesis [64]. Although a common tendency in NO_3_^-^ uptake decrease was observed after flowering in the four NO_3_^-^ treatments, there were quite marked differences in NO_3_^-^ uptake levels between treatments. Thus, immediately after flowering when NO_3_^-^ availability became non-limiting, NO_3_^-^ uptake ranked inversely with the level of pre-flowering NO_3_^-^ treatment. Also, NO_3_^-^ uptake rate at flowering and its dynamics during the first phase immediately afterwards, are in line with a convergence towards a common value for [N]. This observation supports the idea that NO_3_^-^ uptake could be regulated also by plant N status. The high levels of NO_3_^-^ uptake at this time observed in the low-NO_3_^-^ treated plants, was associated with an increase in total plant [N] and this mainly through N enrichment of the existing organs. This phenomenon is a familiar response of plants re-supplied with N after a period of N starvation. Under such conditions increased N uptake rates have been reported in both wheat [65] and barley [24]. Furthermore, the plant [N] convergence between treatments was not due solely to increased plant [N] in the low-NO_3_^-^ treatments but also to decreased plant [N] in the high-NO_3_^-^ treatments. This was linked mainly with a greater increase in stem biomass in the high-NO_3_^-^ treatments than in the low-NO_3_^-^ treatments (data not shown). The latter was probably because of a higher cover density resulting in more light competition among the plants [52]. Because stems are poorer in N than leaves, the relative change in proportion between leaf and stem coupled with a low NO_3_^-^ uptake rate resulted in a decrease in [N] at the whole plant level.

In contrast to the observations of Oscarson *et al*. [16], NO_3_^-^ uptake in our study stopped completely for a period of about 100°Cdays around GS65+300DD to GS65+400DD. Physiologically, this phenomenon coincided with the absence of a significant sink for N. This is because the vegetative parts of the plant were fully developed, while the reproductive parts (the grains) had not yet started active filling. Furthermore, plant [N] was already extremely high at this stage in all treatments.

Still in agreement with the hypothesis of the control of N uptake by the N growth demand, the NO_3_^-^ uptake resumed when the grains started to fill. If grain storage protein filling starts around GS65+240DD, then its maximal rate occurs from about GS65+300DD to GS65+600DD [66]. In absence of any net N remobilisation from the vegetative parts, this phase of renewed NO_3_^-^ uptake was the main source of N for grain filling. Using ^15^N pulse labelling in a hydroponic study, Oscarson *et al*. [17] showed that where N was limiting, a temporary increase in NO_3_^-^ availability late during grain filling resulted in a preferential allocation of newly-assimilated N to the grain. The authors concluded that ^15^N taken up during grain filling was rapidly incorporated in the mobile N pool of the plant and was allocated partly to tissue maintenance but mainly to grain filling. Surprisingly, our different treatments which resulted in highly-contrasting grain yields instead had closely-similar NO_3_^-^ uptake rates during this third phase. This suggests that NO_3_^-^ uptake was slowed in the high-NO_3_^-^ treatments although these, at least potentially, had greater sink demand for N. As NO_3_^-^ was in excess, the ability of plants to assimilate N probably reached its limits under such extreme conditions.

### Relationships between plant NO_3_^-^ uptake and the expression of N metabolism genes

The relative expressions of the seven main genes involved in NO_3_^-^ uptake and N assimilation were followed for the N4 and N10 treatments. These genes are hypothesised to be representative of N network activity and composed of genes coding NO_3_^-^ transporters, and key enzymes involved in NO_3_^-^ reduction and assimilation. Relative expression dynamics were followed in the roots during the post-flowering period with the double purpose (a) of better understanding their behaviour and interrelationships for plants of different N status under non-limiting conditions, and (b) of observing the relationship between ^15^N-based measurements NO_3_^-^ uptake and the expression the N network genes. Although the results presented here for genes expression were focused on root samples, the conclusions of the study should not be biased for two main reasons. Firstly, a large part of N reduction and assimilation occurs in roots during grain filling in wheat (60 to 75% depending on N fertilization level) [67]. Secondly, the high positive correlations observed for all studied genes between roots and flag leaves (S3 Fig), except *TaNADH-GOGAT*, suggest that NO_3_^-^ reduction and assimilation processes could be subjected to a common regulation in these two major organs for N metabolism.

The significant correlation between NO_3_^-^ uptake and *TaNRT2*.*1* relative expression in N4 plants during the post-flowering period suggests an important role for this high-affinity nitrate transporter in NO_3_^-^ uptake. Nevertheless, the absence of a correlation in N10 plants, suggests that the relation is not linear at constant high NO_3_^-^ availability. Conversely, no correlation between NO_3_^-^ uptake and *TaNRT1* relative expression could be detected, suggesting a limited role for this gene under our conditions. Moreover, our results show that *TaNRT2*.*1* is expressed at higher levels than *TaNRT1* despite the high [NO_3_^-^] (10 mM) in the nutrient solution. Comparable results have been found in *Arabidopsis* grown in 10 mM NO_3_^-^ supplied for 5 min with 6 mM NO_3_^-^, where *AtNRT2*.*1* was expressed at higher levels than *AtNRT1*.*1* both in the plant’s vegetative and reproductive stages [21]. Similarly, in maize, *ZmNRT2*.*1* and *ZmNRT2*.*2* were expressed at higher levels than *ZmNRT1*.*1* and *ZmNRT1*.*2* throughout the life cycle with both 0.5 and 2.5 mM NO_3_^-^, and *ZmNRT2*.*1* expression patterns showed strong similarities to the N uptake dynamic [40]. In wheat, Wang *et al*. [36] also showed that *TaNRT2*.*1* is always expressed at higher levels than *TaNRT1* in NO_3_^-^ ranges comparable to ours. These results suggest that *TaNRT2*.*1* participates in NO_3_^-^ uptake in a substantial way, even at high [NO_3_^-^].

Corroborating correlations with NO_3_^-^ uptake, *TaNRT2*.*1* relative expression also correlated with *TaNR* relative expression in roots (Fig. 7A) as well as in flag leaves (S5 Fig). The presumed major role of *TaNRT2*.*1* in post-flowering NO_3_^-^ uptake is supported by its consistent expression dynamics with the rest of the network. Conversely, the expression dynamics of *TaNRT1* was not correlated with *TaNR* and, therefore neither with the rest of the network. *TaNRT2*.*1* thus appears to have a major role in NO_3_^-^ uptake, especially during periods of rapid uptake under non-limiting NO_3_^-^ conditions. However, the correlation between expression and uptake was not perfect, suggesting that *TaNRT2*.*1* is complemented by other NO_3_^-^ transporters operating under a different control mechanism.

Regulation of NO_3_^-^ transporters is a key factor for understanding NO_3_^-^ uptake and NO_3_^-^ assimilation. Several hypotheses have been proposed for the regulation of the NRT2 gene family by internal feedback. According to literature sources, putative inhibitory signals are NO_3_^-^ [24–26] or circulating amino acids such as glutamine [27,28]. Under our conditions, *TaNRT2*.*1* expression was clearly negatively correlated with [NO_3_^-^] in roots, suggesting a negative feedback exerted by the root NO_3_^-^ level. According to this hypothesis, during plant growth, N demand exercised by newly-formed N sinks could limit NO_3_^-^ accumulation in roots, thus allowing the maintenance or increase of *TaNRT2*.*1* expression. Decreases in N sink strength could lead to [NO_3_^-^] increases in the roots, resulting in *TaNRT2*.*1* repression, and NO_3_^-^ uptake decrease. This hypothesis for NO_3_^-^ uptake regulation fits with the assumption that NO_3_^-^ uptake is controlled by plant growth rate under non-limiting conditions.

Study of the relative expressions of the key N network genes reveals their apparent common regulation during post-flowering period, with strong correlations between the various components. Root [NO_3_^-^] would seem to be a good candidate for the role of transmitting the plant N status signal to the NO_3_^-^ transporters. Although relative gene expression studies should be complemented by enzymatic activity assays, because additional regulation may occur at the post-transcriptional level, such as for NR [68], cytosolic GS1 [69], or the root ammonium transporter AMT1.1 [70], our results still seem to indicate a major role for TaNRT2.1 transporter in post-flowering NO_3_^-^ uptake under non-limiting NO_3_^-^ conditions. Plants have shown the ability to quickly regulate their [N] under non-limiting NO_3_^-^ conditions when previously NO_3_^-^ starved, coinciding with increases in the relative expressions of key N metabolism genes. It is possible that this approach may allow identification of genetic variability in the ability to quickly assimilate NO_3_^-^ when NO_3_^-^ is available. A large capacity for NO_3_^-^ assimilation during short favourable periods during the post-flowering growth phase may be a determinant element for increasing grain [N].

### Hypotheses for grain N content limitation

The main justification of studying the physiological basis of post flowering N uptake is its strong link with wheat grain [N], a major agronomic trait. Following the work of Monaghan *et al*. [6], Bogard *et al*. [8] demonstrated that genetic variability in grain [N] was strongly related to post-flowering N uptake capacity, regardless of plant N status at flowering. Thus, one objective of this work was to open tracks in the understanding of the complex regulations of post-flowering N uptake and N assimilation.

Here, high levels of grain yield were associated with decreased grain [N]. This observation is classic, since it is the basis of the extremely well known negative relationship between grain yield and grain protein [4,22,71]. It is, however, less intuitive under our conditions of non-limiting NO_3_^-^ availability during grain filling.

In an ear-halving experiment with four cultivars of contrasting yield potential, Martre *et al*. [72] showed that grain [N] was mainly source-limited as grain [N] increased in halved ears, and as grain N per ear was nearly constant. More precisely, they stated that if yield is clearly limited by sink size, then their ear-halving experiment suggests that grain N filling is relatively more limited by N source. Following this hypothesis, several potential processes limiting N grain filling can be identified, from root N uptake to protein synthesis within the grain, passing through N assimilation and N transport to ears. Our study suggests that NO_3_^-^ uptake can reasonably be dismissed from this series, because of the high post-flowering availability of NO_3_^-^. In addition the results obtained by Oscarson *et al*. [16] show that intrinsic root N uptake capacity was never a limiting factor under hydroponic conditions. Grain protein synthesis capacity and N transport into the ear can also be eliminated. First, there is no obvious reason for a higher capacity for protein synthesis in grains under a low-NO_3_^-^ treatment than in those under a high-NO_3_^-^ treatment, based on the fact that in our study, the grains of the four treatments had comparable individual masses (S4 Table). Second, using wheat ears grown in liquid culture, Barlow *et al*. [73] show that ear capacity to transport N compounds and grain capacity to synthesise protein were not limiting elements. This is supported by the conclusions of Martre *et al*. [72] which state that the capacity of the sink to synthesise proteins does not regulate grain N accumulation. We therefore conclude that, the most likely hypothesis to explain the decrease in grain [N] with increasing yield, is a limitation operating at the level of N assimilation, with a possible saturation of the assimilation pathway.

In our study, increased grain yield was principally related to higher grain numbers per square meter (S4 Table), and these were mainly conditioned by pre-flowering NO_3_^-^ availability. Grain growth rate has been shown to be independent of N availability [74]. The independence between grain development and N availability implies that N demand by the grain during the post-flowering period depends mainly on a plant yield potential established before flowering and therefore varies with the availability of N before flowering. Thus, plants from the low-NO_3_^-^ treatments developed a relatively small yield potential, and filled a low number of grains under later high-NO_3_^-^ conditions after flowering. Conversely, plants from the high-NO_3_^-^ treatments developed a relatively high yield potential, and filled a higher number of grains under the later high-NO_3_^-^ conditions. In the latter case, this necessarily implies a reduced share of available N per grain, thus leading to lower grain [N].

Our results do not allow a precise conclusion regarding the main metabolic process(es) responsible for source limitation. Nevertheless, Jenner *et al*. [75] proposed that a single, or a few, of the amino-acids necessary for grain protein synthesis may limit the global N flux to the grain. For example proline is a major component of storage proteins but represents only a small fraction of the total amino-acid pool in the plant and this can be limiting. In addition, Howarth *et al*. [74] showed that glutamine was the principal amino acid accumulated in the grain, and that its accumulation occurred in the first seven days of grain filling. It may therefore be hypothesised that this key amino-acid may be limiting in the context of a strong demand over a short period of time.

## Conclusion

This study based on a semi-hydroponic culture in controlled conditions of wheat cv. Récital shows that post-flowering NO_3_^-^ uptake is controlled by N status at flowering in early stages following flowering. Latter in the cycle, during the active grain development phase, NO_3_^-^ uptake appeared to be regulated by N demand for growth, although this study did not allow to functionally demonstrate this hypothesis. *TaNRT2*.*1* seems to play a major role in NO_3_^-^ uptake, with expression patterns in both N4 and N10 treatments that were positively correlated with those of the main genes involved in NO_3_^-^ reduction and assimilation. This study also shows that root [NO_3_^-^] could play an important role in the regulation of *TaNRT2*.*1* expression. These findings were obtained on wheat plants at a developmental stage and with a canopy structure that are both meaningful for the underlying agronomic question asked. We believe these results form a good working basis for future research on genetic variability associated with the control of post-flowering N uptake with a long-term goal of forcing a break in the negative relationship between grain protein and grain yield.

## Supporting Information

S1 Dataset Raw data used in the study. (XLSX)

S1 Fig Correlations of NO_3_^-^ concentration between (A) roots and stems and (B) roots and flag leaves for two contrasted NO_3_^-^ treatments (N4 and N10) during the post-flowering period. Values originate from two contrasted N treatments (N4 and N10) at eight post-flowering sampling dates, each including three individual biological repetitions. Statistical analyses were by the Pearson correlation test.
(TIF)

S2 Fig NO_3_^-^ concentration dynamics during the post-flowering period for two contrasting NO_3_^-^ treatments (N4 and N10) in stems (A) and flag leaves (B). Values are the means of four biological repetitions ± 1 standard error for N4 (grey triangles) and N10 (black diamonds).
(TIF)

S3 Fig Correlations of the relative expression levels of five genes implied in NO_3_^-^ reduction and assimilation between roots and flag leaves for two contrasted NO_3_^-^ treatments (N4 and N10) from flowering (GS65) to GS65+800DD. Presented relations are for *TaNR* (**A**), *TaNiR* (**B**), *TaGS2* (**C**), *TaFd-GOGAT* (**D**) and *TaNADH-GOGAT* (**E**). Values originate from seven post-flowering sampling dates, each including three individual biological repetitions for N4 and N10. Gene expression quantification was performed by qRT-PCR. Relative expression values were calculated using the ΔCT method corrected for primers efficiencies, using *Ta54280* and *Ta54948* as internal controls. Statistical analyses were by the Pearson correlation test.
(TIF)

S4 Fig Correlations of the relative expression levels of five genes implied in NO_3_^-^ reduction and assimilation between roots and stems for two contrasted NO_3_^-^ treatments (N4 and N10) from flowering (GS65) to GS65+800DD. Presented relations are for *TaNR* (**A**), *TaNiR* (**B**), *TaGS2* (**C**), *TaFd-GOGAT* (**D**) and *TaNADH-GOGAT* (**E**). Values originate from seven post-flowering sampling dates, each including three individual biological repetitions for N4 and N10. Gene expression quantification was performed by qRT-PCR. Relative expression values were calculated using the ΔCT method corrected for primers efficiencies, using *Ta54280* and *Ta54948* as internal controls. Statistical analyses were by the Pearson correlation test.
(TIF)

S5 Fig Relations between *TaNRT2*.*1* relative expression in roots and *TaNR* relative expression in flag leaves for two contrasted NO_3_^-^ treatments (N4 and N10) from flowering (GS65) to GS65+800DD. Values originate from two contrasted N treatments (N4 and N10) at seven post-flowering sampling dates, each including three individual biological repetitions. Gene expression quantification was performed by qRT-PCR. Relative expression values were calculated using the ΔCT method corrected for primers efficiencies, using *Ta54280* and *Ta54948* as internal controls. Statistical analyses were by the Pearson correlation test.
(TIF)

S1 Table Elemental composition of nutrient solutions adapted from Castle and Randall (1987). (PDF)

S2 Table Target gene name, accession number and primer sequences of primer couples used in the study. (PDF)

S3 Table Roots, stems and flag leaves NO_3_^-^ concentration at flowering for two contrasted NO_3_^-^ treatments (N4 and N10). Presented values are the mean of four biological repetitions ± 1 standard error (SE). Statistically non-significantly different groups (Tukey multiple comparisons, p < 0.05) are labeled with the same lowercase letter.
(PDF)

S4 Table Grain number and thousand-kernel-weight at maturity for the four NO_3_^-^ treatments. Presented values are the mean of four biological repetitions ± 1 standard error (SE). Statistically non-significantly different groups (Tukey multiple comparisons, p < 0.05) are labeled with the same lowercase letter.
(PDF)
