# Supplementary figures and images for: Post-Flowering Nitrate Uptake in Wheat Is Controlled by N Status at Flowering, with a Putative Major Role of Root Nitrate Transporter NRT2.1
Source: PLoS One. 2015 Mar 23;10(3):e0120291. doi: 10.1371/journal.pone.0120291 (PMC4370649; doi:10.1371/journal.pone.0120291)

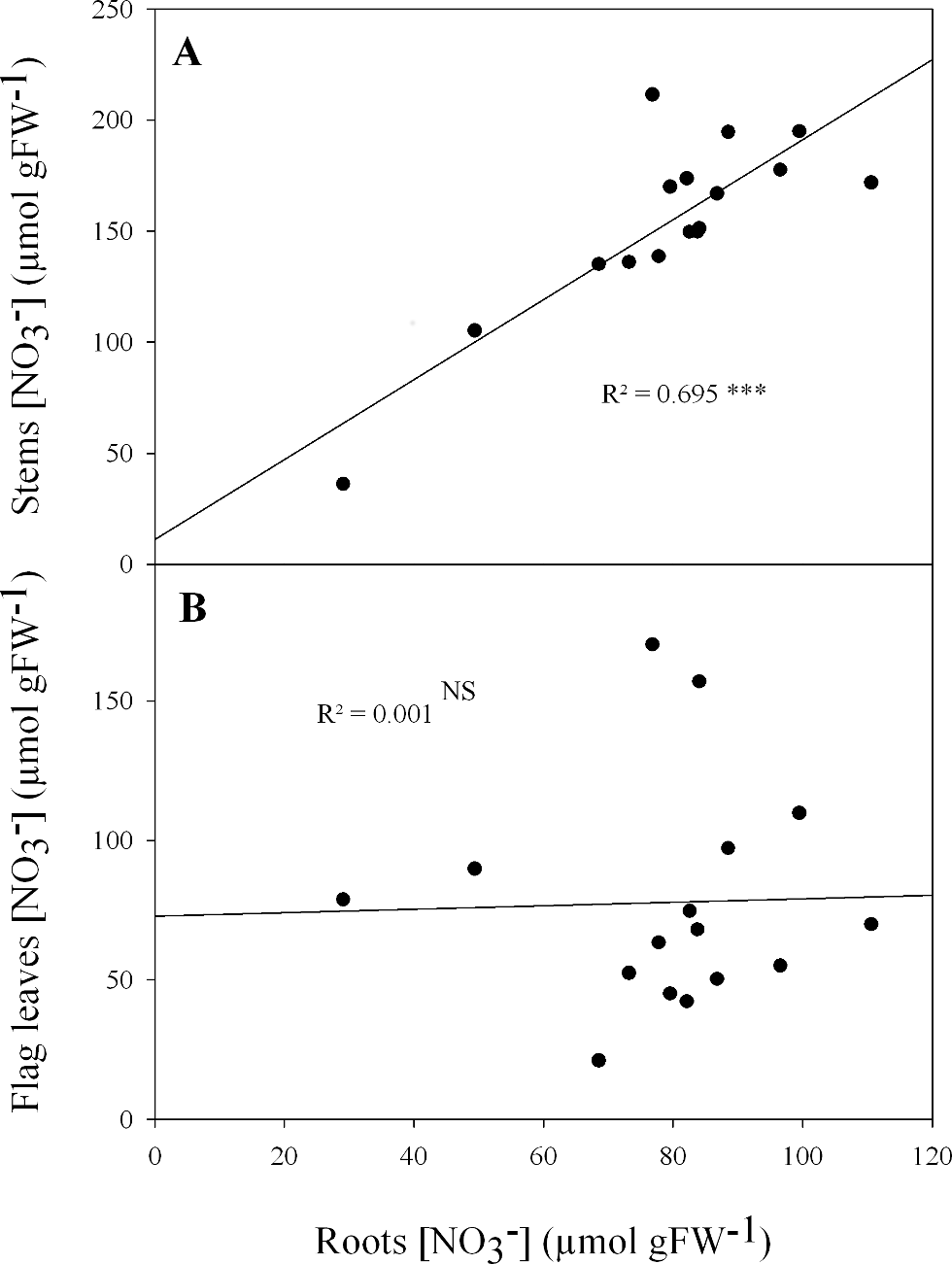

Supplement: S1 Fig — Values originate from two contrasted N treatments (N4 and N10) at eight post-flowering sampling dates, each including three individual biological repetitions. Statistical analyses were by the Pearson correlation test. (TIF) [file pone.0120291.s002.TIF]

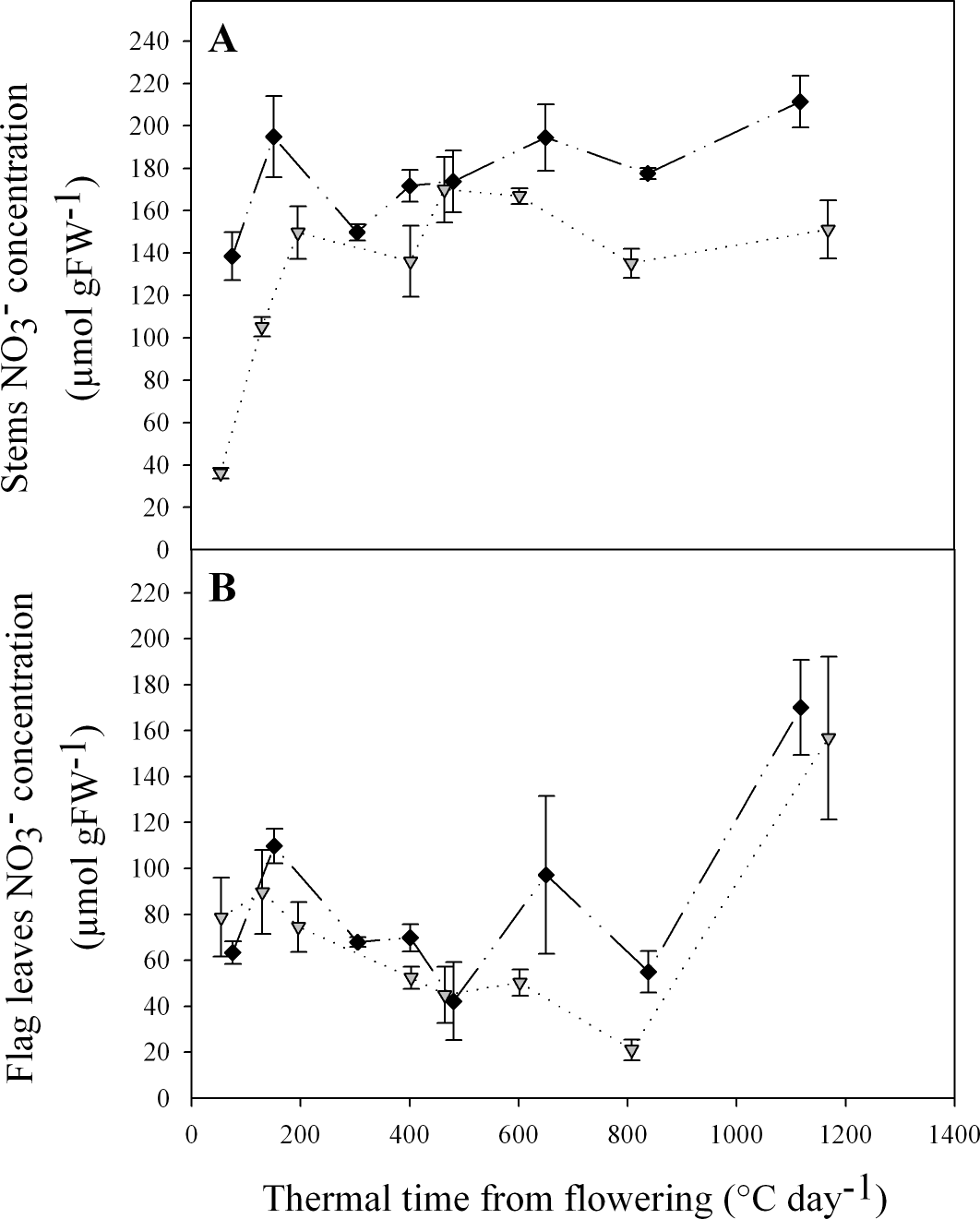

Supplement: S2 Fig — Values are the means of four biological repetitions ± 1 standard error for N4 (grey triangles) and N10 (black diamonds). (TIF) [file pone.0120291.s003.TIF]

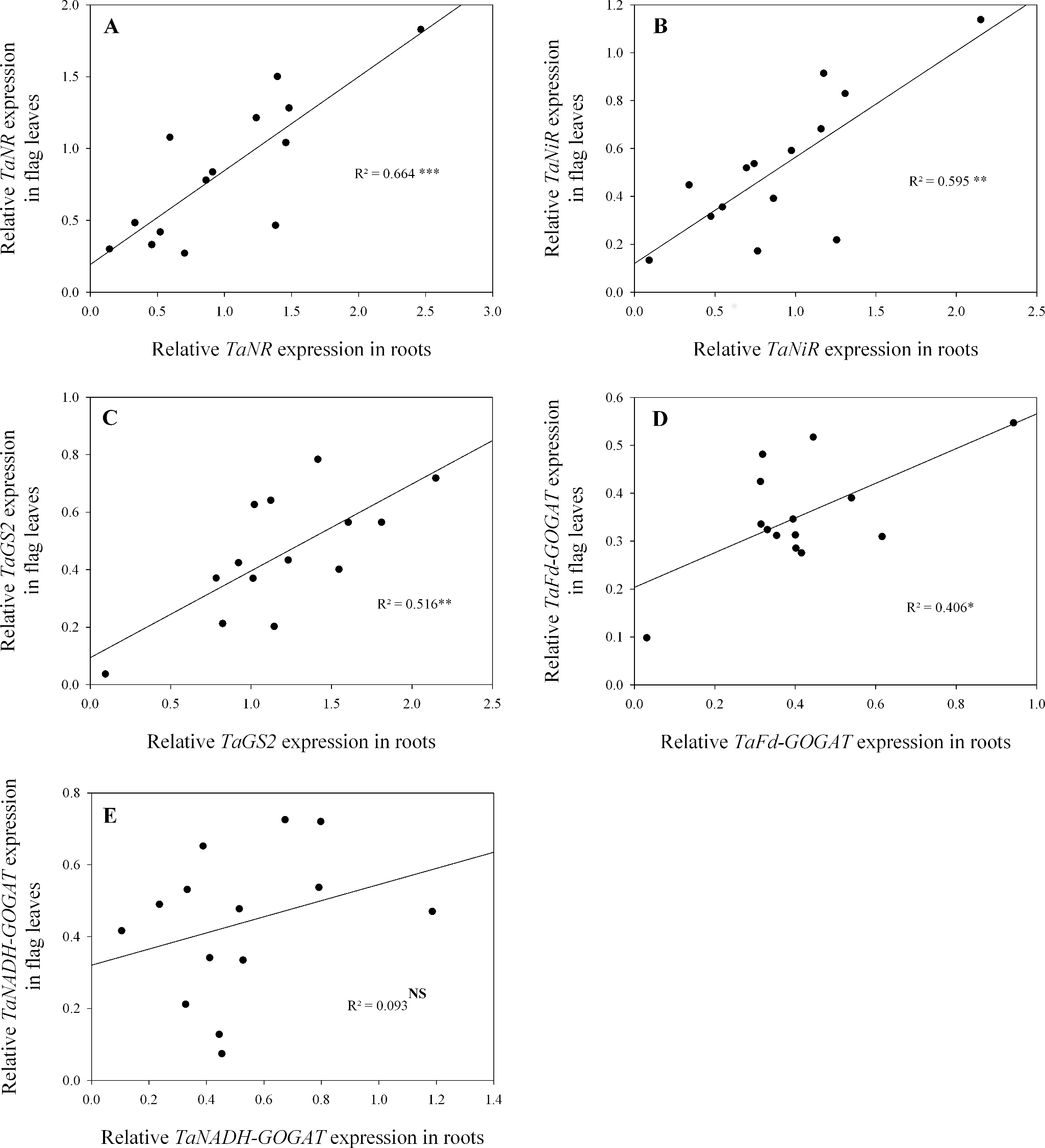

Supplement: S3 Fig — Presented relations are for TaNR (A), TaNiR (B), TaGS2 (C), TaFd-GOGAT (D) and TaNADH-GOGAT (E). Values originate from seven post-flowering sampling dates, each including three individual biological repetitions for N4 and N10. Gene expression quantification was performed by qRT-PCR. Relative expression values were calculated using the ΔCT method corrected for primers efficiencies, using Ta54280 and Ta54948 as internal controls. Statistical analyses were by the Pearson correlation test. (TIF) [file pone.0120291.s004.TIF]

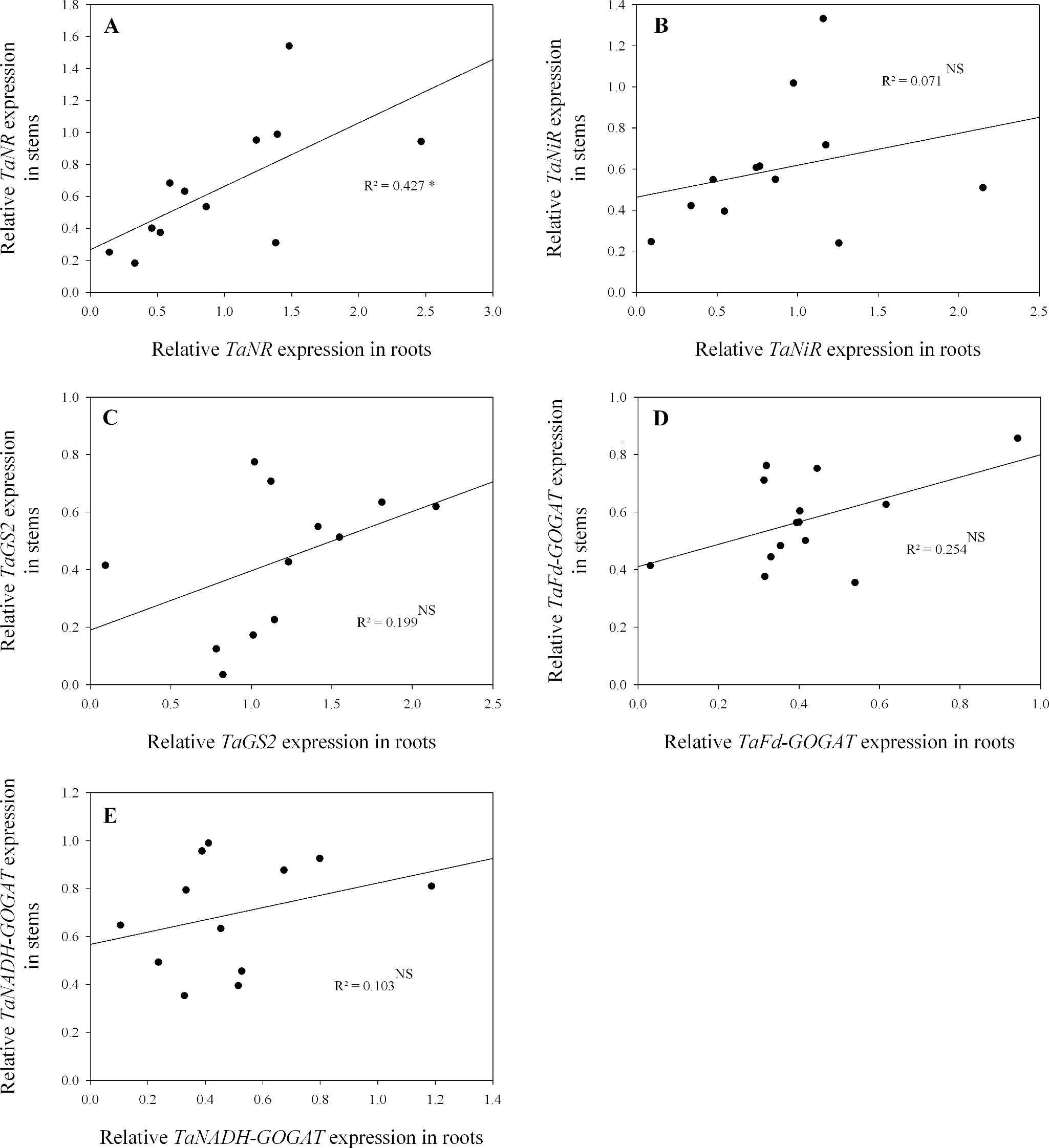

Supplement: S4 Fig — Presented relations are for TaNR (A), TaNiR (B), TaGS2 (C), TaFd-GOGAT (D) and TaNADH-GOGAT (E). Values originate from seven post-flowering sampling dates, each including three individual biological repetitions for N4 and N10. Gene expression quantification was performed by qRT-PCR. Relative expression values were calculated using the ΔCT method corrected for primers efficiencies, using Ta54280 and Ta54948 as internal controls. Statistical analyses were by the Pearson correlation test. (TIF) [file pone.0120291.s005.TIF]

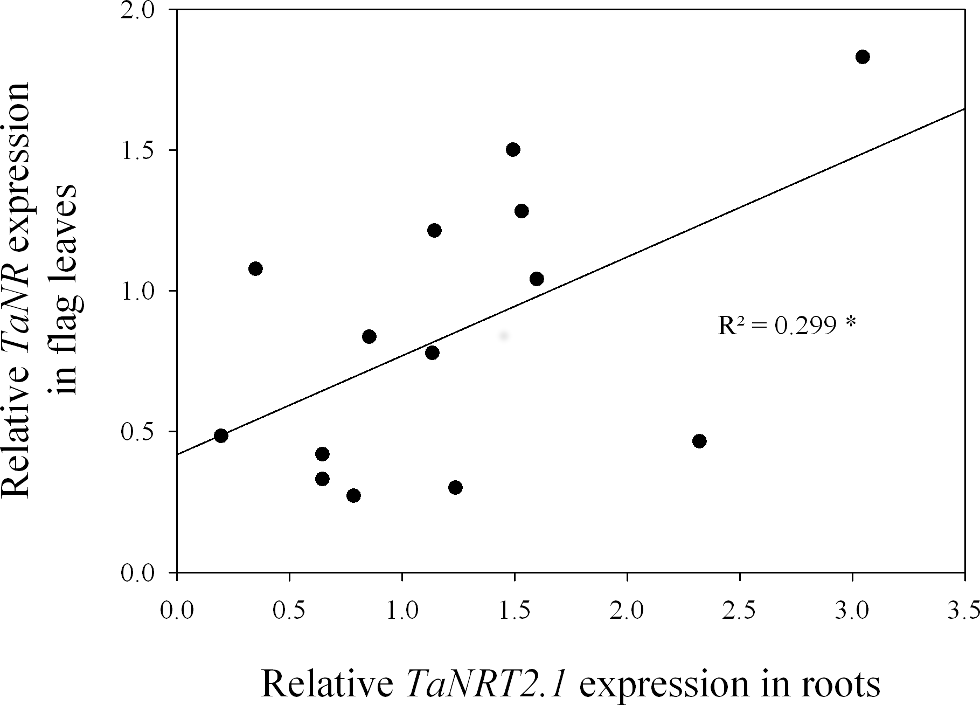

Supplement: S5 Fig — Values originate from two contrasted N treatments (N4 and N10) at seven post-flowering sampling dates, each including three individual biological repetitions. Gene expression quantification was performed by qRT-PCR. Relative expression values were calculated using the ΔCT method corrected for primers efficiencies, using Ta54280 and Ta54948 as internal controls. Statistical analyses were by the Pearson correlation test. (TIF) [file pone.0120291.s006.TIF]
