# Supplementary material for: Post-Flowering Nitrate Uptake in Wheat Is Controlled by N Status at Flowering, with a Putative Major Role of Root Nitrate Transporter NRT2.1
Source: PLoS One. 2015 Mar 23;10(3):e0120291. doi: 10.1371/journal.pone.0120291 (PMC4370649; doi:10.1371/journal.pone.0120291)
Supplement: S1 Table — (PDF) [file pone.0120291.s007.pdf]

		N Treatment			
		**N1**	**N4**	**N7**	**N10**
**Macroelements (mM)**	**KH_2PO_4**	1,0	1,0	1,0	1,0
	**KNO_3**	0,0	3,0	4,0	5,0
	**$Ca(NO_3)_2$, $4H_2O$**	0,5	0,5	1,5	2,5
	**$MgSO_4$, $7H_2O$**	2,0	2,0	2,0	2,0
	**$CaCl_2$, 2HO**	3,0	3,0	3,0	2,0
	**KCl**	5,0	2,0	1,0	0,0
**Microelements (µM)**	**H_3BO_3**	10,0	10,0	10,0	10,0
	**$ZnCl_2$**	0,7	0,7	0,7	0,7
	**$CuCl_2$, $2H_2O$**	0,4	0,4	0,4	0,4
	**$MnCl_2$, $4H_2O$**	4,5	4,5	4,5	4,5
	**MoO_3**	0,2	0,2	0,2	0,2
	**EDFS-Fe**	50,0	50,0	50,0	50,0
