# Supplementary material for: Post-Flowering Nitrate Uptake in Wheat Is Controlled by N Status at Flowering, with a Putative Major Role of Root Nitrate Transporter NRT2.1
Source: PLoS One. 2015 Mar 23;10(3):e0120291. doi: 10.1371/journal.pone.0120291 (PMC4370649; doi:10.1371/journal.pone.0120291)
Supplement: S2 Table — (PDF) [file pone.0120291.s008.pdf]

**Primer pair name**	**Used for**	**Reference / Genbank accession number / Unigene contig**	**Primer**	**Sequence**
**TaNR**	Nitrate reductase	Boisson *et al.*, 2005	Forward	CGACTGCACCGCCTTCCTCA
			Reverse	CGCCGGCGACCTTGGTG
**TaNiR**	Nitrite reductase	Boisson *et al.*, 2005	Forward	CCCTACACCAACCTCCTCTCCTCC
			Reverse	GTCGTTGATGTGCGGGTGCTC
**TaFd-GOGAT**	Ferredoxin-dependent glutamate synthase	Boisson *et al.*, 2005	Forward	TGGTGCCACCCAGCGAAGA
			Reverse	AGCTCGTTTCCAGAAGATGCCTTG
**TaNADH-GOGAT**	NADH glutamate synthase	Boisson *et al.*, 2005	Forward	CAGTGGTCCTGCTGGTTTGGCT
			Reverse	TGCATTGTTCTCAGAACGGAGACG
**TaGS2**	Glutamine synthetase 2	DQ124212.1	Forward	CATACTACTGCGCCGTAGGATCAGAC
			Reverse	GGCATGACCTCCCCGTTTGTT
**TaNRT1**	Nitrate transporter NRT1	AY587264	Forward	GAGCTGAGGAATCACATGGCAAAAC
			Reverse	CATGAAGGCTGGCTCTGGGGT
**TaNRT2.1**	Nitrate transporter NRT2.1	AF332214.1	Forward	GCCGCTTGTCTTCCACGCA
			Reverse	GTCCTTGGCCATGTCTCCCTTCT
**Ta54280**	Translation initiation factor SuI1 family protein	Ta.54280	Forward	TCTGCTGTAATGGTACTGTAGTC
			Reverse	TCCGTGAATCTTGATGCTCTC
**Ta54948**	Rab GTPase homolog (Rab7 subfamily)	Ta.54948	Forward	GAACGAGCCAGAGGAAGAAC
			Reverse	AAATACGGGTCCACAAGTCAC
