# Supplementary material for: Post-Flowering Nitrate Uptake in Wheat Is Controlled by N Status at Flowering, with a Putative Major Role of Root Nitrate Transporter NRT2.1
Source: PLoS One. 2015 Mar 23;10(3):e0120291. doi: 10.1371/journal.pone.0120291 (PMC4370649; doi:10.1371/journal.pone.0120291)
Supplement: S3 Table — Presented values are the mean of four biological repetitions ± 1 standard error (SE). Statistically non-significantly different groups (Tukey multiple comparisons, p < 0.05) are labeled with the same lowercase letter. (PDF) [file pone.0120291.s009.pdf]

NO_3^- treatment	Roots [NO_3^-] (µmol g^{-1}) ±SE	Stems [NO_3^-] (µmol g^{-1}) ±SE	Flag leaves [NO_3^-] (µmol g^{-1}) ±SE
**N4**	29.09 ± 4.22 *b*	36.16 ± 2.49 *b*	78.78 ± 17.15 *a*
**N10**	77.78 ± 7.41 *a*	138.58 ± 11.42 *a*	63.37 ± 4.87 *a*
