# Supplementary material for: Post-Flowering Nitrate Uptake in Wheat Is Controlled by N Status at Flowering, with a Putative Major Role of Root Nitrate Transporter NRT2.1
Source: PLoS One. 2015 Mar 23;10(3):e0120291. doi: 10.1371/journal.pone.0120291 (PMC4370649; doi:10.1371/journal.pone.0120291)
Supplement: S4 Table — Presented values are the mean of four biological repetitions ± 1 standard error (SE). Statistically non-significantly different groups (Tukey multiple comparisons, p < 0.05) are labeled with the same lowercase letter. (PDF) [file pone.0120291.s010.pdf]

**N treatment**	**Grain number (per m^{-2}) ±SE**	**Thousand Kernel Weight (g) ±SE**
**N1**	22955 ± 3443 *a*	26,24 ± 0,77 *a*
**N4**	30759 ± 3108 *a*	29,91 ± 1,45 *ab*
**N7**	50253 ± 2407 *b*	31,75 ± 1,08 *b*
**N10**	58808 ± 4836 *b*	28,74 ± 0,64 *ab*
